# A Weighted Voting Approach for Traditional Chinese Medicine Formula Classification Using Large Language Models: Algorithm Development and Validation Study

**DOI:** 10.2196/69286

**Published:** 2025-07-24

**Authors:** Zhe Wang, Keqian Li, Suyuan Peng, Lihong Liu, Xiaolin Yang, Keyu Yao, Heinrich Herre, Yan Zhu

**Affiliations:** 1 Institute of Basic Medical Sciences, Chinese Academy of Medical Sciences; School of Basic Medicine, Peking Union Medical College, Beijing, China; 2 Institute for Medical Informatics, Statistics and Epidemiology, University of Leipzig, Leipzig, Germany; 3 School of Medical Information, Changchun University of Chinese Medicine, Changchun, China; 4 Institute of Information on Traditional Chinese Medicine, China Academy of Chinese Medical Sciences, No 16, Nanxiao Street, Dongzhimen, Beijing, 100010, China, 86 010 64089639; 5 Institute for Computer Science, University of Leipzig, Leipzig, Germany

**Keywords:** traditional Chinese medicine, TCM formula classification, large language models, ensemble learning, algorithm development

## Abstract

**Background:**

Several clinical cases and experiments have demonstrated the effectiveness of traditional Chinese medicine (TCM) formulas in treating and preventing diseases. These formulas contain critical information about their ingredients, efficacy, and indications. Classifying TCM formulas based on this information can effectively standardize TCM formulas management, support clinical and research applications, and promote the modernization and scientific use of TCM. To further advance this task, TCM formulas can be classified using various approaches, including manual classification, machine learning, and deep learning. Additionally, large language models (LLMs) are gaining prominence in the biomedical field. Integrating LLMs into TCM research could significantly enhance and accelerate the discovery of TCM knowledge by leveraging their advanced linguistic understanding and contextual reasoning capabilities.

**Objective:**

The objective of this study is to evaluate the performance of different LLMs in the TCM formula classification task. Additionally, by employing ensemble learning with multiple fine-tuned LLMs, this study aims to enhance classification accuracy.

**Methods:**

The data for the TCM formula were manually refined and cleaned. We selected 10 LLMs that support Chinese for fine-tuning. We then employed an ensemble learning approach that combined the predictions of multiple models using both hard and weighted voting, with weights determined by the average accuracy of each model. Finally, we selected the top 5 most effective models from each series of LLMs for weighted voting (top 5) and the top 3 most accurate models of 10 for weighted voting (top 3).

**Results:**

A total of 2441 TCM formulas were curated manually from multiple sources, including the Coding Rules for Chinese Medicinal Formulas and Their Codes, the Chinese National Medical Insurance Catalog for proprietary Chinese medicines, textbooks of TCM formulas, and TCM literature. The dataset was divided into a training set of 1999 TCM formulas and test set of 442 TCM formulas. The testing results showed that Qwen-14B achieved the highest accuracy of 75.32% among the single models. The accuracy rates for hard voting, weighted voting, weighted voting (top 5), and weighted voting (top 3) were 75.79%, 76.47%, 75.57%, and 77.15%, respectively.

**Conclusions:**

This study aims to explore the effectiveness of LLMs in the TCM formula classification task. To this end, we propose an ensemble learning method that integrates multiple fine-tuned LLMs through a voting mechanism. This method not only improves classification accuracy but also enhances the existing classification system for classifying the efficacy of TCM formula.

## Introduction

### Background

Traditional Chinese medicine (TCM) formulas are combinations of medicinal substances developed through clinical experience and guided by TCM theory. They are developed through a systematic process involving syndrome differentiation, etiological analysis, determination of therapeutic principles, selection of appropriate herbs, dosage adjustment, formulation considerations, and specification of use, all in accordance with fundamental compositional structures [1]. Several studies have shown that TCM formulas have significant efficacy in the treatment and prevention of disease in both clinical and experimental studies [2-4]. A randomized clinical trial demonstrated that the TCM compound Tongxinluo significantly improved clinical outcomes in patients diagnosed with ST-segment elevation myocardial infarction [5]; the traditional medicine Pien Tze Huang prevents colorectal cancer by influencing the gut microbiota, enhancing beneficial metabolites, and suppressing oncogenic and proinflammatory factors [6].

Classical TCM formulas represent exemplary applications of TCM prescriptions. The selection and cataloging system for classical formulas streamlines the approval process for Chinese herbal compound preparations by exempting them from pharmacological studies and clinical trial data submissions. These formulas are defined as “those derived from ancient classical prescriptions that remain widely used today, demonstrate proven efficacy, exhibit distinctive characteristics and advantages, and were documented in medical texts prior to or during the Qing Dynasty.” However, after thousands of years of clinical practice, TCM has accumulated an immense number of formulas by the late Qing period, incomplete statistics indicate that over 100,000 had been recorded [7]. Current TCM formula textbooks and national standards still follow the efficacy-oriented classification system established in Wang Ang’s Qing Dynasty work *Yi Fang Ji Jie* (Compilation of Medical Formulas, 医方集解) [8]. Prior to the introduction of this system, many formulas lacked clear categorization, making manual classification both labor-intensive and susceptible to inconsistencies.

As the foundation of TCM syndrome differentiation and treatment, systematic classification of TCM formulas based on efficacy enables a comprehensive exploration of their latent information and reveals herb-disease relationship patterns. Previous research primarily relied on expert experience to develop classification systems organized by therapeutic methods (efficacy) [9,10]. With advances in artificial intelligence, new methodological pathways have emerged for automated formula classification. Gu [11] used the k-nearest neighbor algorithm in machine learning to calculate and classify the similarity of TCM formulas. However, the complex relationships between herbs and diseases in TCM formulas complicate the classification process. Cheng et al [12] present an improved deep learning model: S-TextBLCNN for the TCM formula classification task; it has an accuracy of 0.858 and an *F*_1_-score of 0.762. To further explore the optimal deep learning models in the TCM formula classification task, Ren et al [13] combined several deep learning models to classify TCM formulas and found that bidirectional encoder representation from transformers-convolutional neural network was the most effective, achieving an accuracy of 77.87%, as well as weighted precision, weighted recall, and weighted *F*_1_-score of 79.46%, 77.87%, and 77.44%, respectively. In 2022, OpenAI released applications such as ChatGPT [14], have demonstrated strong performance in tasks like question answering and translation. Moreover, large language models (LLMs) have achieved remarkable results in the biomedical domain. For instance, Google’s Med-PaLM2, which was fine-tuned from PaLM2 using data from the medical domain, achieved 86.5% accuracy on the MedQA dataset [15], which is close to the response level of clinical doctors [16]. Nijkamp et al [17] have trained the ProGen2 model on many different sequence datasets and demonstrated state-of-the-art performance both in generating novel viable protein sequences and in predicting protein fitness tasks. Some scholars tried to use LLMs to classify the text [18,19], therefore, to verify the classification ability of LLMs. In our previous study [20], we used the prompt templates and LLMs, such as ChatGLM-6B [21], ChatGLM2-6B [22], InternLM-20B [23], ChatGLM-130B [24], and ChatGPT, to classify TCM formula and validate the potential of LLMs in the field of TCM.

Based on our previous study [20], this study further explores the classification of TCM formulas by fine-tuning 10 distinct LLMs. It introduces an ensemble voting method based on multiple fine-tuned LLMs. This approach emphasizes the integration of predictions from each model through voting mechanisms, including both hard voting and weighted voting. The adoption of multiple LLMs for TCM formula classification offers a novel perspective on the application of LLMs in the field of TCM.

### Research Question

In our previous work [20], we used LLMs to investigate TCM formula classification. We used prompt templates and both fine-tuned and original LLMs. The experimental results demonstrated that fine-tuned LLMs can enhance classification task accuracy. Therefore, in this study, we aim to explore the potential of LLMs in TCM formula classification tasks and rationality by posing the following research questions (RQs):

- RQ1: What is the performance of different LLMs for TCM formula classification?
- RQ2: How to improve the performance of TCM formula classification by multiple LLMs?
- RQ3: What is the rationality of TCM formula classification using LLMs?

To explore RQ1, we used 10 LLMs and fine-tuned them on a manually curated TCM formula dataset, followed by a comparative analysis of the results. To address RQ2, we used an ensemble learning approach. Using the average accuracy derived from the fine-tuned LLMs as model weights, we performed both hard and weighted voting on the results. In addition, we explored the effectiveness of different strategies, including selecting the best-performing model within each category and weighted voting based on the 3 best-performing models, to determine the approach that yields optimal results. To investigate RQ3, TCM experts manually reviewed the predicted results generated by the fine-tuned LLMs. This process involved analyzing discrepancies between the voting results of the LLMs and the reference answer, thereby exploring the rationality behind the classification by the LLMs in the TCM formula classification task.

## Methods

### Data Preparation

We used 2441 TCM formulas manually curated from the Coding Rules for Chinese Medicinal Formulas and Their Codes [25], the Chinese National Medical Insurance Catalog for proprietary Chinese medicines (2023) [26], and textbooks of formulas of Chinese medicine [10]. After manual review [13], each formula consists of the formula name, ingredients, efficacy, and indications. The data processing procedure is as follows: only efficacy-related classifications are retained, while non-efficacy classifications, such as ethnic minority medicine, are removed; formulas under identical or similar classifications are consolidated (with classification names standardized according to national guidelines), while unique formula classifications are preserved; finally, the resulting data are subjected to deduplication.

In the Qing Dynasty, Wang Ang, in his work *Yifang Jijie*, proposed an integrated classification method that prioritized the efficacy of the TCM formula. This method was not only used in the textbooks of Chinese medicine formulas throughout various dynasties [27] and the secondary classification of the Chinese National Medical Insurance Catalog for proprietary Chinese medicines [28] but also served as the method used in the national standard, the Coding Rules for Chinese Medicinal Formulas and Their Codes. Therefore, this study established a harmonized efficacy classification system based on national standards, integrating multisource TCM formula data through structural realignment and category consolidation (the data remain intact with only standardized nomenclature adjustments made to classification categories exhibiting terminological variations, following national regulatory requirements), resulting in 22 standardized categories. However, The emetic formulations category contained insufficient samples and was therefore excluded; based on this categorization, a total of 2441 formulations were identified across 21 categories, as detailed in Table 1.

**Table 1.** Statistical information of traditional Chinese medicine formula data.

Standard efficacy	Abbreviation	Chinese name	Formulations (N=2441), n (%)
Supplementing and boosting formula	SBF	补益剂	417 (17.08)
Heat-clearing formula	HCF	清热剂	411 (16.84)
Blood-regulating formula	BRF	理血剂	372 (15.24)
Desiccating formula	DF	祛湿剂	246 (10.08)
Superficies relieving formula	SF	解表剂	160 (6.55)
Resolving phlegm relieving cough and relieving wheezing formula	RPRCRWF	化痰-止咳-平喘剂	160 (6.55)
Qi regulated formula	QRF	理气剂	134 (5.49)
Formula for wind disorder	FWD	治风剂	119 (4.86)
Warming interior formula	WIF	温里剂	73 (2.99)
Formula for purgation	FP	泻下剂	60 (2.46)
Reconciling formula	RF	和解剂	43 (1.76)
Tranquillization formula	TF	安神剂	39 (1.60)
Digestive formula	DIF	消食剂	38 (1.56)
Astringent formula	AF	固涩剂	32 (1.31)
Softening hard lumps and dispelling nodes formula	SHLDNF	消肿散结剂	27 (1.11)
Formula for treating carbuncle and ulcer	FTCU	痈疡剂	25 (1.02)
Summer-heat-expelling formula	SHEF	祛暑剂	22 (0.90)
Formula for resuscitation	FR	开窍剂	21 (0.86)
Antidryness formula	ADF	治燥剂	16 (0.66)
Resolving turbidity and lowering lipids formula	RTLLF	化浊降脂剂	16 (0.66)
Antihelminthic formula	AHF	驱虫剂	10 (0.41)

### Selected LLMs

Since TCM formulas are documented in Chinese or ancient Chinese, in this study, the LLMs that have exceptional performance in Chinese or support multilingualism were selected to facilitate the model’s understanding of this information [29].

#### ChatGLM

ChatGLM-6B, ChatGLM2-6B, and ChatGLM3 are a series of open bilingual language models developed by the Knowledge Engineering Group and Data Mining of Tsinghua University. These models can be easily deployed and fine-tuned on standard consumer-grade graphics processing units, enabling users to perform personalized tasks [21,22,29]. The official script was used to fine-tune these LLMs in this study.

#### InternLM

The Shanghai Artificial Intelligence Laboratory, SenseTime Technology, the Chinese University of Hong Kong, and Fudan University jointly introduced InternLM-20B and InternLM-7B, which showed exceptional performance in areas such as mathematics, code, dialogue, and creative writing [23]. In this study, Xtuner [30] was used to fine-tune InternLM-20B for TCM formula classification. This tool helps users to fine-tune LLMs with limited hardware resources.

#### Baichuan2

Baichuan2 is a multilanguage LLM developed by Baichuan-AI. It has been trained on 2.6 trillion tokens and performs well in medical and legal areas. Currently, Baichuan2 has released 7B and 13B to users [31]. We fine-tuned Baichuan2-7B and Baichuan2-13B for our task using Xtuner.

#### Qwen

Qwen is a series of language models introduced by AliCloud, including Qwen-1.8B, Qwen-7B, Qwen-14B, and Qwen-72B [32]. The official script was used to fine-tune these models in this study. It is worth noting that we fine-tuned Qwen-1.8B with full parameters to improve the performance of the task.

#### BLOOM

BigScience [33] has launched a series of models of different sizes, known as the BigScience Large Open-Science Open-Access Multilingual Language Model (BLOOM), including 1B, 7B, 13B, and 176B. BLOOM’s LLM generates text in multiple languages and codes. In this study, we fine-tuned the BLOOM-1.7B with full parameters using the LLMTuner tool [34].

### Ensemble Voting Algorithms for TCM Formula Classification

In our prior work, we used methods with prompt templates and fine-tuned LLMs to classify TCM formulas. The fine-tuned ChatGLM2-6B demonstrated optimal performance, achieving an accuracy rate of 71% in the classification task. However, other models did not surpass the 70% accuracy rate. Its performance failed to surpass that of the deep learning model trained on a single task. In a different study, Chatterjee et al [35] proposed that an ensemble voting method outperforms traditional single classifiers in accurately diagnosing Alzheimer disease. To improve the accuracy of the fine-tuned LLMs in the TCM formula classification task, we adopted an ensemble learning approach, this methodology involved using several fine-tuned LLMs and developing a voting mechanism specifically tailored to their outputs. The goal of this methodology is to combine the predictions of multiple models thereby increasing the accuracy of the TCM formula classification task.


(1) $$y_{\text{hard}}=\arg\max_{c}\sum_{i=0}^{N}1(C_{i}=c)$$

In equation 1, we have *N* fine-tuned LLMs, where the predicted value for LLM_*i*_ is denoted by $C_{i}$ , *N*=10, and *c* represents all classes of the TCM formula. $1\left(C_{i}=c\right)$ is the indicator function, equal to 1 if $C_{i}=c$, and 0 otherwise. The final prediction result $y_{hard}$ is determined by selecting the class with the highest number of votes.


(2) $$y_{\text{weighted}}=\arg\max_{c}\sum_{i=0}^{N}\alpha_{i}1(C_{i}=c)$$

In equation 2, $\alpha_{i}$ represents the average accuracy of each LLM, the votes for each LLM are multiplied by the corresponding weights, and the resulting weighted votes are summed. Finally, the TCM formula class with the highest weighted sum is finally selected as the final prediction.

### Evaluation Metrics

To evaluate the performance of the fine-tuned LLMs on text classification tasks, we used the accuracy, precision, recall, and *F*_1_-score, as defined in equation 3-6, based on true positives (TP), true negatives (TN), false positives (FP), and false negatives (FN).


(3) $$Accuracy=\frac{TP+TN}{TP+TN+FP+FN}$$

(4) $$\begin{array}{c} Precision=\frac{TP}{TP+FP} \end{array}$$

(5) $$Recall=\frac{TP}{TP+FN}$$

(6) $$F_{1}\text{-}score=\frac{2\cdot recall\cdot precision}{recall+precision}$$

Due to the unbalanced distribution of the TCM formula in our test set, we calculated the weighted precision, the weighted recall, and the weighted *F*_1_-score to comprehensively evaluate the performance of the models in multiclassification tasks in equation 7-9.


(7) $${Precision}_{\text{weighted-avg}}=\sum_{i=1}^{L}\left({Precision}_{i}\cdot \omega_{i}\right)$$

(8) $${Recall}_{\text{weighted-avg}}=\sum_{i=1}^{L}\left({Recall}_{i}\cdot \omega_{i}\right)$$

(9) $${F_{1}\text{-}score}_{\text{weighted-avg}}=\sum_{i=1}^{L}\left({F_{1}\text{-}score}_{i}\cdot \omega_{i}\right)$$

With the weighted approach, the performance in detecting classes with a larger number of samples can be adequately represented by assigning weights based on the percentage of sample numbers. In our current investigation, we are dealing with a dataset containing 21 classes of TCM formulas. In equation 7-9, *L*=21, where $\omega_{i}$ is the proportion of each TCM formula in the total dataset.

### Experiment

#### Experimental Design and Verification

##### Overview

**Figure 1.**
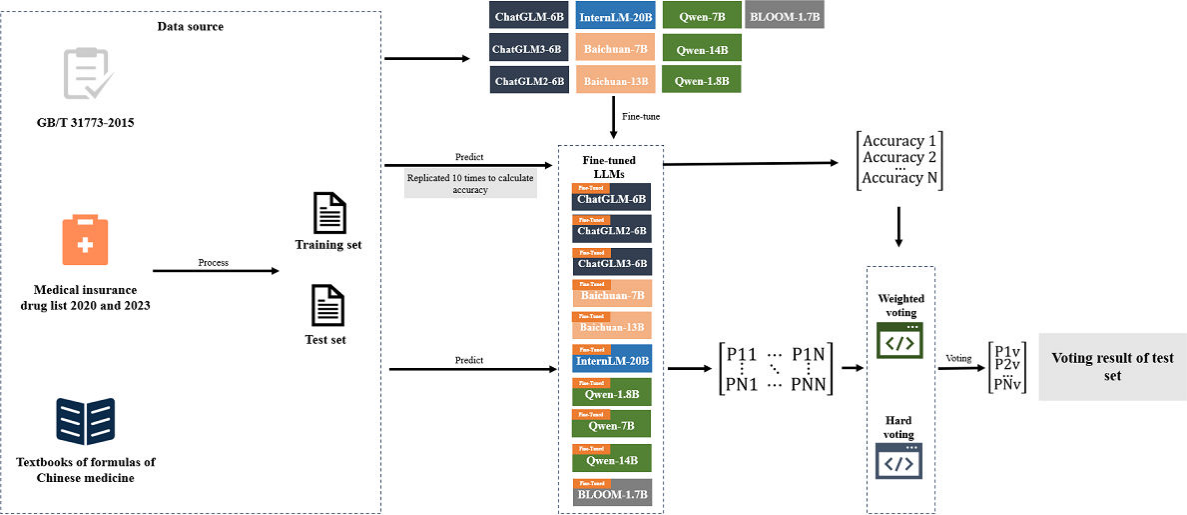
Workflow of traditional Chinese medicine formula classification using large language models. BLOOM: BigScience Large Open-Science Open-Access Multilingual Language Model.

We designed the following experiment to explore the RQ1, RQ2, and RQ3, as shown in Figure 1.

##### Experiment 1

To address RQ1, a prompt dataset was formulated from the curated TCM formula data. The processed prompt dataset was then divided into a training set and a test set containing 1999 and 442 formulas, respectively. The selected LLMs were fine-tuned using the training set, and their performance was evaluated using the test set. This process was iterated 10 times to obtain the average accuracy.

##### Experiment 2

To validate RQ2, we used a multistage validation process. First, we implemented a hard voting approach to aggregate model predictions and derive a consolidated result. We then calculated the accuracy of this method. In the next step, we assigned weights to each model based on its average accuracy. Using a weighted voting strategy, we then calculated the accuracy of the weighted voting method. In the third step, we selected the model with the highest accuracy from each category and used these selections for weighted voting. Finally, we identified the top 3 models among the fine-tuned LLMs and conducted a weighted vote to determine the most effective voting method.

##### Experiment 3

To validate RQ3, we extracted a subset of data from the voting results that met the following two conditions:(1) All model predictions were identical but different from the reference answer, or (2) more than 80% of the model predictions were the same but different from the reference answer. TCM experts subsequently reviewed this subset to evaluate the rationality of the LLMs’ classification results.

### Experimental Configuration Parameter and Platform

To ensure robust performance, we fine-tuned LLMs using the common configurations and selected the model that achieved the most stable and optimal results. The details of the configurations for the fine-tuned LLMs are shown in Multimedia Appendix 1. The fine-tuning and verification were done by running and validating the LLMs on graphics processing unit computer servers.

### Ethical Considerations

We confirm that this study did not involve human or animal subjects. It used publicly available, deidentified text data and sourced from the web. Therefore, no ethics approval was required in accordance with relevant institutional guidelines and the JMIR editorial policy on ethics review requirements.

## Results

### The Experimental Results of Each Fine-Tuned LLM

**Figure 2.**
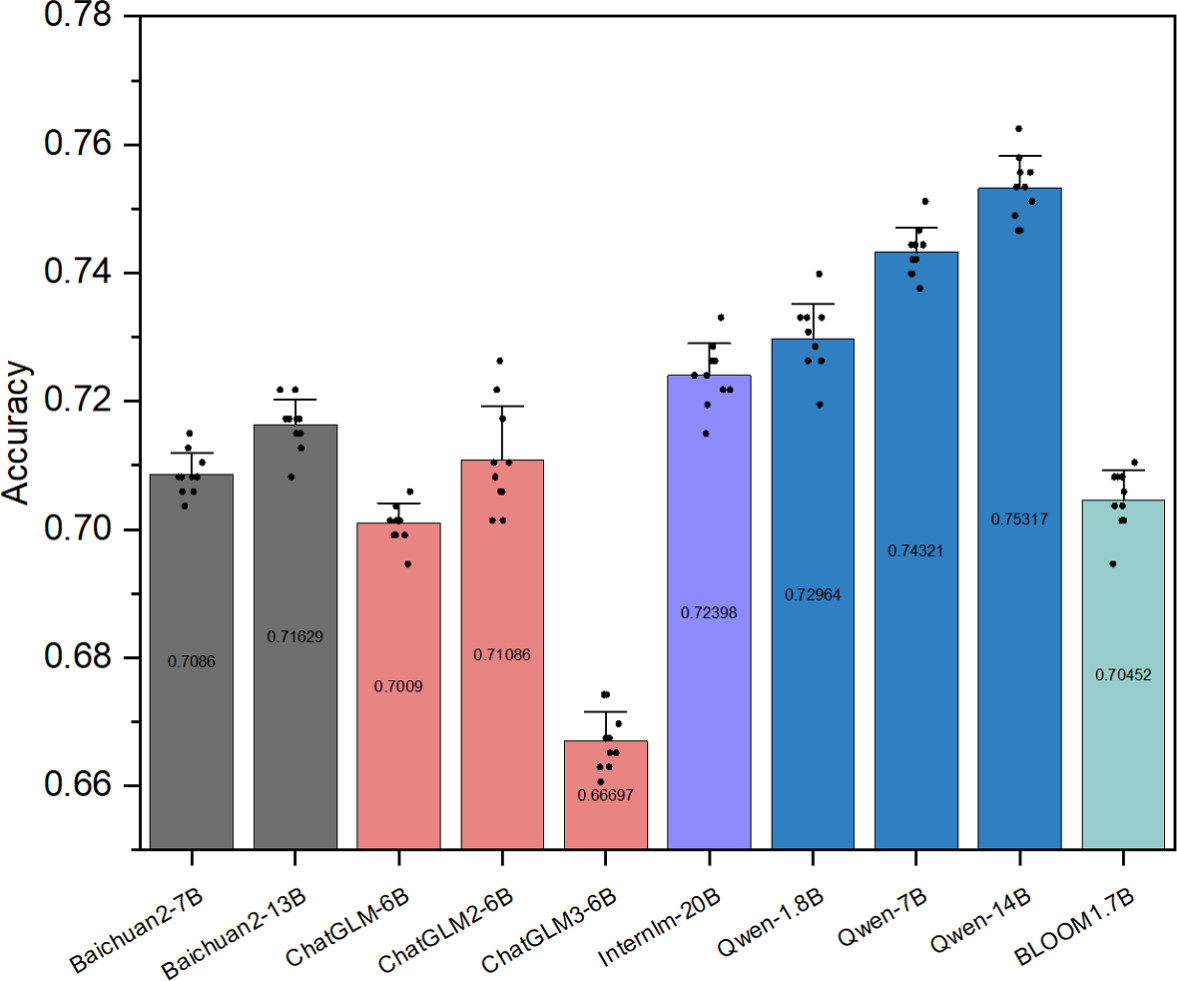
The average accuracy of each large language model in experiment 1. BLOOM: BigScience Large Open-Science Open-Access Multilingual Language Model.

In experiment 1, we performed a 10-iteration validation for each model using the test set. Figure 2 shows the mean accuracy for each model. Notably, Qwen-14B (mean 75.32%, SD 0.48%) and Qwen-7B (mean 74.32%, SD 0.37%) showed the highest performance. Close behind were Qwen-1.8B (mean 72.96%, SD 0.53%), InternLM-20B (mean 72.40%, SD 0.47%), Baichuan2-7B (mean 70.86%, SD 0.32%), Baichuan2-13B (mean 71.63%, SD 0.38%), ChatGLM-6B (mean 70.09%, SD 0.30%), ChatGLM2-6B (mean 71.09%, SD 0.80%), and BLOOM-1.7B (mean 70.45%, SD 0.44%), all with accuracies above 70%. However, ChatGLM3-6B (mean 66.70%, SD 0.44%) did not exceed 70% accuracy (Table 2). The fine-tuning of individual LLMs does not lead to remarkable results for the TCM formula classification task in this study. It is noteworthy that the Qwen series of LLMs showed promising performance in the TCM classification task, and Qwen-1.8B and BLOOM-1.7B achieved significant accuracy after full-parameter fine-tuning.

**Table 2.** The experimental results of each fine-tuned large language model (LLM).

LLMs	Accuracy (%), mean (SD)
Qwen-14B	75.32 (0.48)
Qwen-7B	74.32 (0.37)
Qwen-1.8B	72.96 (0.53)
InternLM-20B	72.40 (0.47)
Baichuan2-7B	70.86 (0.32)
Baichuan2-13B	71.63 (0.38)
ChatGLM-6B	70.09 (0.30)
ChatGLM2-6B	71.09 (0.80)
ChatGLM3-6B	66.70 (0.44)
BLOOM-1.7B	70.45 (0.44)

### TCM Formula Classification Using Ensemble Learning

**Figure 3.**
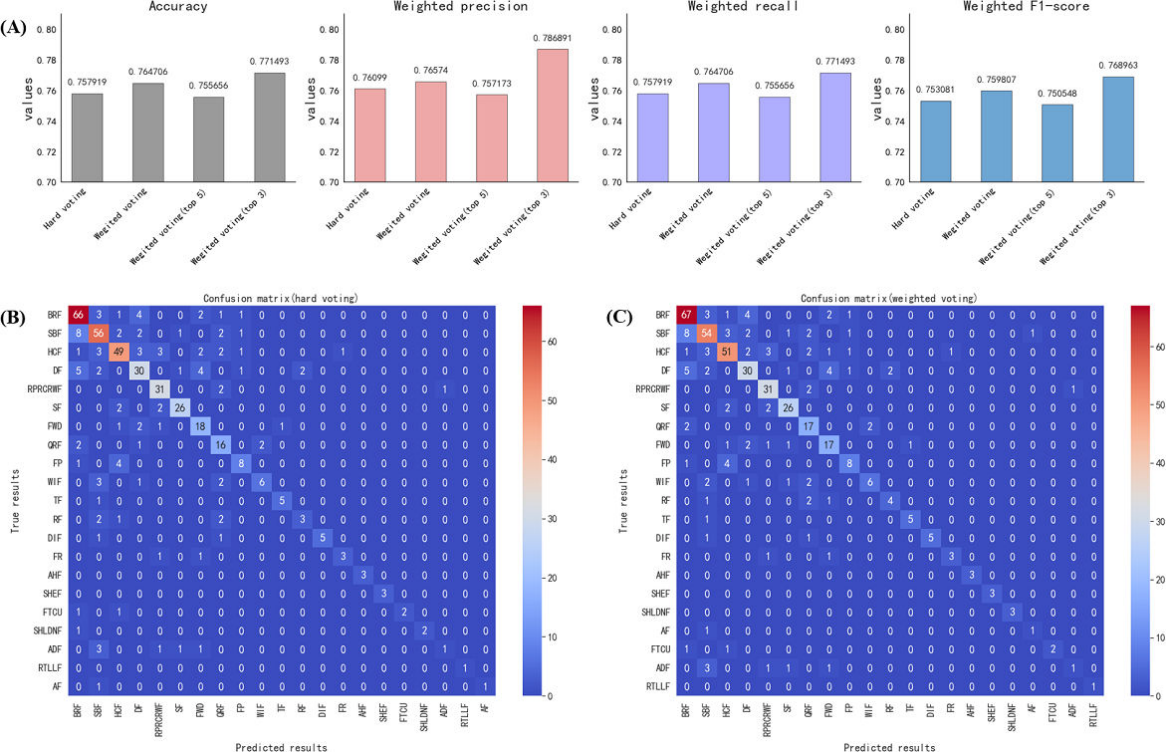
Accuracy, weighted precision, weighted recall, and weighted *F*_1_-score and their confusion matrices in experiment 2. (A) Accuracy, weighted precision, weighted recall, and weighted *F*_1_-score of different ensemble voting methods. (B) Confusion matrix of the hard voting method. (C) Confusion matrix of the weighted voting method. ADF: antidryness formula; AF: astringent formula; AHF: antihelminthic formula; BRF: blood-regulating formula; DF: desiccating formula; DIF: digestive formula; FP: formula for purgation; FR: formula for resuscitation; FTCU: formula for treating carbuncle and ulcer; FWD: formula for wind disorder; HCF: heat-clearing formula; QRF: Qi regulated formula; RF: reconciling formula; RPRCRWF: resolving phle-m relieving cough and relieving wheezing formula; RTLLF: resolving turbidity and lowering lipids formula; SBF: supplementing and boosting formula; SF: superficies relieving formula; SHEF: summer-heat-expelling formula; SHLDNF: softening hard lumps and dispelling nodes formula; TF: tranquilization formula; WIF: warming interior formula.

In experiment 2, to improve the accuracy of the classification task for validating RQ2, we used an ensemble learning approach by integrating fine-tuned LLMs for collective voting predictions. Given the clear result type of our fine-tuned model results (Table 3), we first applied hard voting, which resulted in an accuracy of 75.79%. The weighted precision, weighted recall, and weighted *F*_1_-scores were calculated as 76.10%, 75.79%, and 75.31%, respectively. The confusion matrix is shown in Figure 3B. These results outperformed those of the single models; however, due to the generally low accuracy of our fine-tuned models, the single model results could potentially influence the final results. We therefore developed a weighted voting method using the average accuracy of each model as the weight. This approach resulted in an accuracy of 76.47%, with weighted precision, weighted recall, and weighted *F*_1_-scores of 76.57%, 76.47%, and 75.98%, respectively. The weighted voting results not only outperformed direct hard voting but also showed superior performance on the 21 classifications within the test set, as shown in Figure 3C.

**Table 3.** Findings of experiment 2.

Method	Accuracy (%)	Weighted precision (%)	Weighted recall (%)	Weighted *F*_1_- score (%)
Hard voting	75.79	76.10	75.79	75.31
Weighted voting	76.47	76.57	76.47	75.98
Weighted voting (top 5)	75.57	75.72	75.57	75.05
Weighted voting (top 3)	77.15	78.69	77.15	76.90

To further investigate the impact of multiple LLMs on the voting results, we selected the best-performing model within each category (top 5) and performed weighted voting, such as Baichuan-13B, ChatGLM2-6B, InternLM-20B, Qwen-14B, and BLOOM-1.7B. The statistical results showed an accuracy of 75.57%, a weighted precision of 75.72%, a weighted recall of 75.57%, and a weighted *F*_1_-score of 75.05%, as shown in Figure 3A. The confusion matrix is shown in Multimedia Appendix 2.

We selected the 3 models with the highest accuracy, such as Qwen-14B, Qwen-7B, and Qwen-1.8B, to subject their prediction results to weighted voting. The resulting accuracy was 77.15%, the weighted precision was 78.69%, the weighted recall was 77.15%, and the weighted *F*_1_-score was 76.90%, as shown in Figure 3A. The confusion matrix is shown in Multimedia Appendix 3. As can be seen in Figure 3A, the accuracy obtained by weighted voting (top 3) was the highest, surpassing the accuracy obtained by hard voting. Hard voting and weighted voting (top 5) were similar, with no significant differences.

### Results Analysis of Test Set

**Figure 4.**
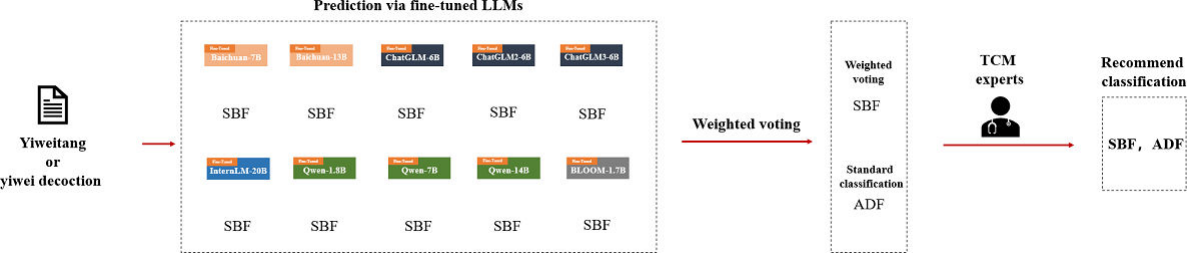
Results analysis of TCM experts. ADF: antidryness formula; LLM: large language model; SBF: supplementing and boosting formula; TCM: traditional Chinese medicine.

During the analysis of LLM voting results on the test set, through TCM expert discussions on the classification results, we observed that in some cases, all LLMs voted the same way, but the results did not match the standard answers. Experts judged that the voting results of the LLMs had a certain degree of rationality. In such cases, it is necessary to discuss why the models reached the same voting results that deviate from the original answers and the rationality of these voting results. We illustrate this with a typical example (Figure 4).

TCM formula “Yiweitang or Yiwei decoction (YWD)” consists of Chinese herbs: Bei Sha Shen, Mai Dong, Bing Tang, Di Huang, and Yu Zhu. The indications for treatment are stomach yin injury syndrome. Symptoms include a burning and dull pain in the stomach, lack of appetite despite hunger, dry mouth and throat, dry and hard stool, dry retching, and hiccups. The existing classification of YWD is antidryness formula. However, current scholars have researched Yiwei decoction for the treatment of premature ovarian insufficiency [36,37] and the prevention of osteoporosis related to it [38], proving its efficacy through methods such as network pharmacology and molecular research. Zhang and Zhu [39] have also used the spectrum-effect relationship and network pharmacology to screen for the antioxidant components of Yiwei decoction, demonstrating its function in nourishing stomach yin. Both the treatment or prevention of premature ovarian insufficiency and its related diseases, as well as the nourishing stomach yin function of YWD, fall under the category of supplementing and boosting formula.

In summary, through these discussion points and specific examples, we can gain a deeper understanding of the performance of LLMs in the classification of TCM formula. This can serve as a basis for exploring and reflecting on the existing classification system of formula efficacy. It provides reference and insights for future research on formula efficacy classification.

## Discussion

### Principal Findings

TCM formulas contain valuable information on ingredients, efficacy, and indications. They serve as important reference for researchers in both clinical and experimental contexts. In this study, we used fine-tuned LLMs in combination with an ensemble learning approach to classify TCM formulas and identify potential information, thereby improved the accuracy of TCM formula classification. Our approach provides a new method for studying formulaic information in TCM.

In this study, we posed 3 RQs and designed corresponding experiments to explore the potential and rationality of fine-tuned LLMs in the TCM formula classification task. The results showed that the fine-tuned Qwen-14B performed remarkably well in the task, achieving an average accuracy of 75.32% (SD 0.48%). However, the accuracy of a single model did not exceed that of deep learning models trained for a single task. To enhance accuracy, we used 2 ensemble methods: hard voting and weighted voting. These methods integrate the 10 fine-tuned LLMs. The accuracy of hard voting and weighted voting reached 75.79% and 76.47%, respectively. The results demonstrate that both hard voting and weighted voting outperform the individual LLMs. Notably, weighted voting (top 3) achieved the highest accuracy, reaching an 77.15%. To evaluate the rationality of the LLMs in the task, we analyzed their prediction errors. In the selected example, all models produced the same prediction, which differed from the reference answer based on YWD. After being analyzed by TCM experts, some of the results predicted by the LLMs were deemed reasonable and can be used as a reference to improve the existing efficacy classification of TCM formulas.

For RQ3, compared to traditional rule-based or deep learning approaches, LLMs demonstrate superior capabilities in automatically identifying latent relationships between herbal formulas. By integrating multidimensional information, such as herbal composition, therapeutic effects, clinical indications, and modern medical research, they build more comprehensive classification systems to uncover potential information. Experimental results show that LLMs can detect potential categories that are not recognized in existing expert classification frameworks. In typical examples, LLM-predicted classifications were validated as clinically plausible by expert panel reviews and literature evidence, suggesting their potential to provide novel scientific foundations for updating and optimizing expert-based classification systems.

Therefore, from both theoretical and technical perspectives, we posit that LLMs can generate more meaningful outcomes for TCM formula classification. This serves as a basis for exploring and reflecting on the existing classification system of formula efficacy, providing references and insights for the subsequent screening of classic formulas.

### Limitations

However, despite numerous studies on the effectiveness of LLMs in classification tasks [40-42], there is a lack of research on text classification in the field of TCM. Our study confirmed that LLMs can achieve a certain effect in the TCM formula classification task. Due to the complexity of TCM knowledge, achieving high accuracy with simple fine-tuning and prompt engineering is difficult. In future research, the composition of herbs in formulas can be encoded through vectors to better explore potential relationships in TCM formula. LLMs in the Qwen series are trained on large-scale, high-quality, and diverse Chinese and English corpora, and have performance across various tasks, in our study, we fully fine-tuned Qwen-1.8B to enhance its capability in classifying TCM formulas and achieved significant accuracy. In the future, we plan to fully fine-tune more LLMs, such as internLM-1.8B [43], and use a weighted voting approach to improve classification accuracy.

Several limitations may have influenced the predicted results. The predictive performance of the LLMs was affected by the relatively small and unevenly distributed dataset of TCM formula in our study. In the future, we plan to collect and curate a large, high-quality TCM formula dataset and integrate it with knowledge graphs to enhance the prediction accuracy of a single LLM. Additionally, we also aim to further improve the performance of the TCM formula classification task by refining our weighted voting methodology.

### Conclusions

This study explored the performance of various fine-tuned LLMs in the TCM formula classification task. To improve classification accuracy, both hard voting and weighted voting methods were employed. In conclusion, we also examined the rationality of using LLMs for TCM formula classification and discussed the potential of improving existing classification standards of TCM formulas through the application of LLMs.

## Supplementary material

10.2196/69286 Multimedia Appendix 1 Configuration parameters for each fine-tuned large language model.

10.2196/69286 Multimedia Appendix 2 Confusion matrix of the weighted voting method with the top 5 models.

10.2196/69286 Multimedia Appendix 3 Confusion matrix of the weighted voting method with the top 3 models.

## Abbreviations

BLOOM: BigScience Large Open-Science Open-Access Multilingual Language Model
FN: false negative
FP: false positive
TN: true negative
TP: true positive
GPUs: Graphics Processing Units
LLM: large language model
RQ: research question
TCM: traditional Chinese medicine
YWD: Yiweitang or Yiwei decoction
