# Supplementary material for: A Weighted Voting Approach for Traditional Chinese Medicine Formula Classification Using Large Language Models: Algorithm Development and Validation Study
Source: JMIR Med Inform. 2025 Jul 24;13:e69286. doi: 10.2196/69286 (PMC12292024; doi:10.2196/69286)
Supplement: Multimedia Appendix 1 [file medinform-v13-e69286-s001.docx]

| **LLMs** | **Fine-tuned parameter** | **Value** |
| --- | --- | --- |
| ChatGLM-6B | Fine-tuned type | P-Tuning v2 |
|  | Learn rate | 2e-2 |
|  | Max step | 4000 |
| ChatGLM2-6B | Fine-tuned type | P-Tuning v2 |
|  | Learn rate | 2e-2 |
|  | Max step | 4000 |
| ChatGLM3-6B | Fine-tuned type | P-Tuning v2 |
|  | Learn rate | 2e-2 |
|  | Max step | 4000 |
| Baichuan-7B | Fine-tuned type | QLoRA |
|  | Learn rate | 2e-4 |
|  | Max epochs | 50 |
| Baichuan-13B | Fine-tuned type | QLoRA |
|  | Learn rate | 2e-4 |
|  | Max epochs | 50 |
| Qwen-1.8B | Fine-tuned type | Full parameter fine-tuning |
|  | Learn rate | 1e-5 |
|  | Train epochs | 50 |
| Qwen-7B | Fine-tuned type | LoRA |
|  | Learn rate | 3e-4 |
|  | Train epochs | 50 |
| Qwen-14B | Fine-tuned type | LoRA |
|  | Learn rate | 3e-4 |
|  | Train epochs | 50 |
| InternLM-20B | Fine-tuned type | QLoRA |
|  | Learn rate | 2e-4 |
|  | Max epochs | 30 |
| Bloom-1.7B | Fine-tuned type | Full parameter fine-tuning |
|  | Learn rate | 2e-5 |
|  | Train epochs | 150 |
