# Supplementary figures and images for: A Weighted Voting Approach for Traditional Chinese Medicine Formula Classification Using Large Language Models: Algorithm Development and Validation Study
Source: JMIR Med Inform. 2025 Jul 24;13:e69286. doi: 10.2196/69286 (PMC12292024; doi:10.2196/69286)

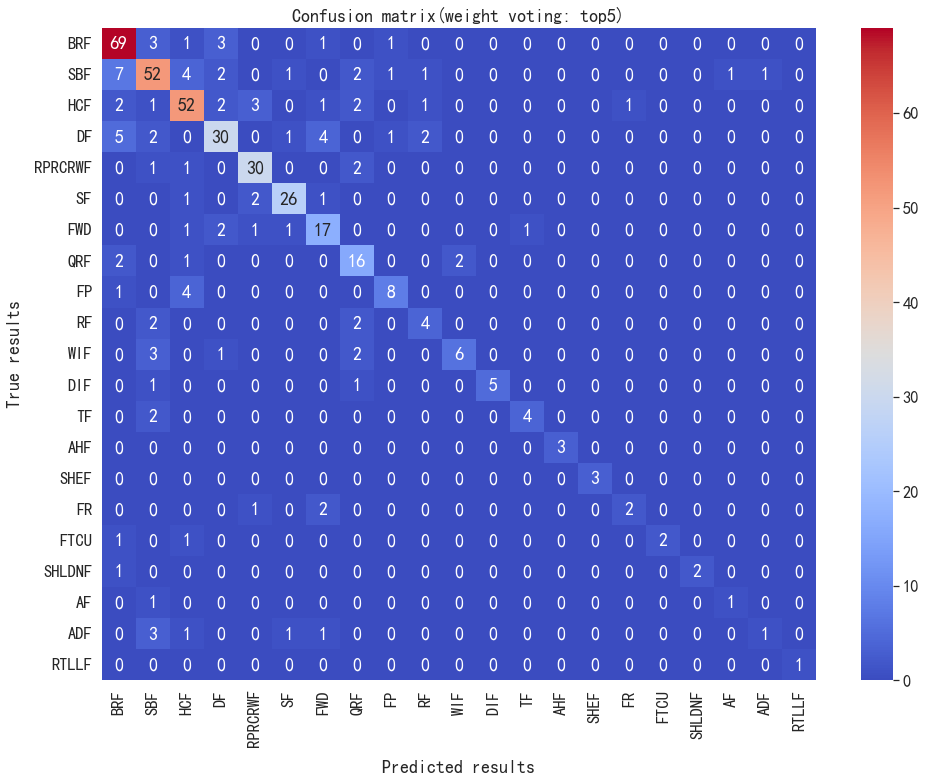

Supplement: Multimedia Appendix 2 [file medinform-v13-e69286-s002.png]

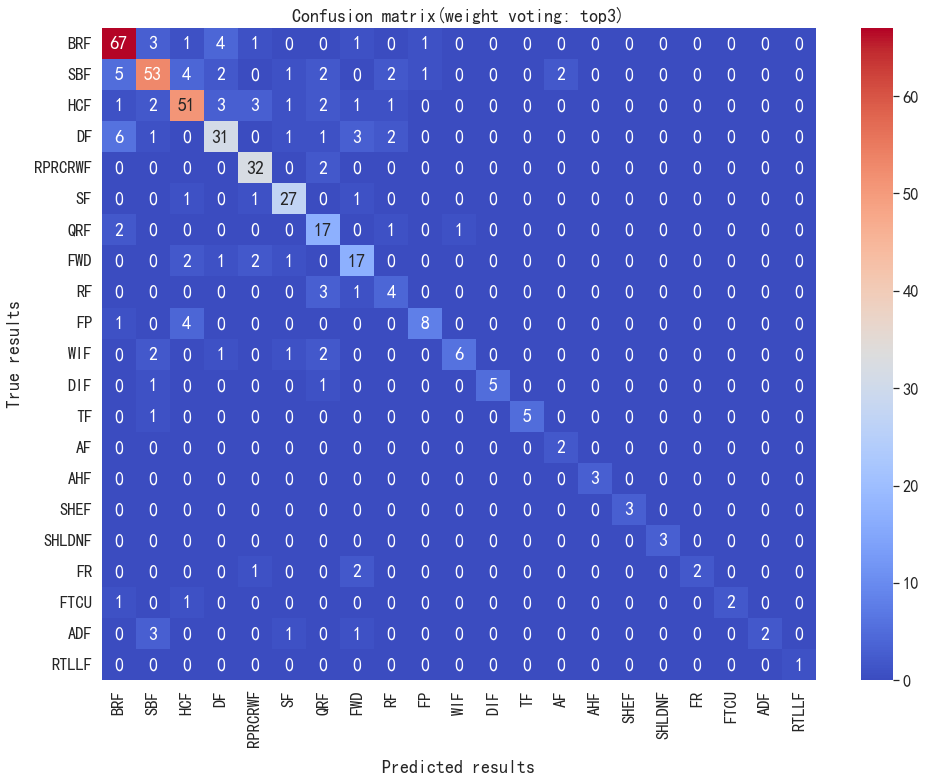

Supplement: Multimedia Appendix 3 [file medinform-v13-e69286-s003.png]
